# Diaphragmatic parameters by ultrasonography for predicting weaning outcomes

**DOI:** 10.1186/s12890-018-0739-9

**Published:** 2018-11-23

**Authors:** Pongdhep Theerawit, Dararat Eksombatchai, Yuda Sutherasan, Thitiporn Suwatanapongched, Charn Kiatboonsri, Sumalee Kiatboonsri

**Affiliations:** 1 Division of Critical Care Medicine, Department of Medicine, Faculty of Medicine Ramathibodi Hospital, Mahidol University, Bangkok, Thailand; 2 Division of Pulmonary and Pulmonary Critical Care Medicine, Department of Medicine, Faculty of Medicine Ramathibodi Hospital, Mahidol University, Bangkok, 270 Rama 6 Road, Thung Phaya Thai, Ratchathewi, Bangkok, 10400 Thailand; 3 Department of Diagnostic and Therapeutic Radiology, Faculty of Medicine Ramathibodi Hospital, Mahidol University, Bangkok, Thailand

**Keywords:** Diaphragm ultrasound, Weaning, Spontaneous breathing trial, Diaphragmatic weakness, Diaphragmatic dysfunction

## Abstract

**Background:**

Diaphragmatic dysfunction remains the main cause of weaning difficulty or failure. Ultrasonographic measurement of diaphragmatic function can be used to predict the outcomes of weaning from mechanical ventilation. Our primary objective was to investigate the performance of various sonographic parameters of diaphragmatic function for predicting the success of weaning from mechanical ventilation.

**Methods:**

We prospectively enrolled 68 adult patients requiring mechanical ventilation who were admitted to the intensive care unit from June 2013 to November 2013. The diaphragmatic inspiratory excursion, time to peak inspiratory amplitude of the diaphragm (TPIA_dia_), diaphragmatic thickness (DT), DT difference (DTD), and diaphragm thickening fraction (TFdi) were determined by bedside ultrasonography performed at the end of a spontaneous breathing trial. A receiver operating characteristic curve was used for analysis.

**Results:**

In total, 62 patients were analyzed. The mean TPIA_dia_ was significantly higher in the weaning success group (right, 1.27 ± 0.38 s; left, 1.14 ± 0.37 s) than in the weaning failure group (right, 0.97 ± 0.43 s; left, 0.85 ± 0.39 s) (*P* <  0.05). The sensitivity, specificity, positive predictive value, and negative predictive value of a TPIA_dia_ of > 0.8 s in predicting weaning success were 92, 46, 89, and 56%, respectively. The diaphragmatic inspiratory excursion, DTD, and TFdi were associated with reintubation within 48 h. The *P* values were 0.047, 0.021, and 0.028, and the areas under the receiver operating characteristic curve were 0.716, 0.805, and 0.784, respectively.

**Conclusion:**

Among diaphragmatic parameters, TPIA_dia_ exhibits good performance in predicting the success of weaning from mechanical ventilation. This study demonstrated a trend toward successful use of TPIA_dia_ rather than diaphragmatic inspiratory excursion as a predictor of weaning from mechanical ventilation.

**Electronic supplementary material:**

The online version of this article (10.1186/s12890-018-0739-9) contains supplementary material, which is available to authorized users.

## Background

Diaphragmatic dysfunction remains the main cause of weaning difficulty or failure. Its prevalence ranges from 33 to 95% [1–5]. Diaphragmatic dysfunction among patients hospitalized in the intensive care unit (ICU) is commonly attributed to critical illness polyneuropathy and myopathy [6]. Mechanical ventilation, even after a short period of time, can also induce diaphragmatic dysfunction by reducing the force that generates the capacity of the diaphragm, which may cause weaning difficulty. Diaphragmatic dysfunction can be worsened by disuse atrophy of both fast-twitch and slow-twitch myofibers of the diaphragm following the administration of a neuromuscular blocking agent [7–9].

Several parameters, including the rapid shallow breathing index (RSBI), vital capacity (VC), and maximum peak inspiratory pressure (PI_MAX_), are routinely used to predict weaning failure from mechanical ventilation. However, the sensitivity, specificity, positive predictive value (PPV), negative predictive value (NPV), and cut-off values of these parameters are highly variable among studies. Moreover, these parameters do not directly reflect diaphragmatic function [10].

Although fluoroscopic examination of the diaphragm remains the gold standard for evaluation of diaphragmatic movement, it cannot be performed in patients in the ICU. Bedside ultrasound is being increasingly performed for real-time assessment of diaphragmatic movement. This technique allows qualitative and quantitative assessment of diaphragmatic function in terms of diaphragmatic thickness (DT) and diaphragmatic amplitude during contraction, which help to diagnose diaphragmatic weakness [11–14] and respiratory workload [12, 15] in patients in the ICU. Kim et al. [16] found that diaphragmatic excursion of < 10 mm or paradoxical movement is associated with weaning failure. DiNino et al. [17] suggested that diaphragm contractile function at the zone of apposition as measured by ultrasound may be useful for predicting extubation success or failure. Spadaro et al. [18] recently reported a new index, namely the diaphragmatic RSBI, which is calculated by substituting the tidal volume (TV) with the ultrasonographic evaluation of diaphragmatic displacement. This parameter is more accurate than the traditional RSBI for prediction of weaning failure [18].

To the best of our knowledge, quantitative assessment of both the right and left hemidiaphragms and comparison of these ultrasound parameters with conventional parameters have not been performed. We hypothesized that diaphragmatic parameters determined by bedside ultrasonography can predict the weaning outcome with better performance than conventional parameters. Therefore, we conducted this prospective study to investigate diaphragmatic function in patients in the ICU in terms of diaphragmatic motion and contractile function using bedside ultrasonography during the weaning period. We also evaluated the performance of these ultrasound parameters by comparison with conventional parameters routinely used to predict successful weaning from mechanical ventilation.

## Methods

### Study design and patient population

This prospective cross-sectional study was conducted at a tertiary-care, university-based hospital. The study was approved by the Ethical Clearance Committee on Human Rights Related to Research Involving Human Subjects, Faculty of Medicine Ramathibodi Hospital, Mahidol University (No. MURA2013/414/N_3_SEP_17_).

We consecutively enrolled adult patients aged > 18 years who were admitted to the medical or surgical ICU of Ramathibodi Hospital, Mahidol University from June 2013 to November 2013. All patients provided written informed consent prior to enrollment. When the patient was unable to provide consent because of consciousness disturbance, the next of kin provided written informed consent.

### Inclusion and exclusion criteria

The inclusion criteria were as follows: 1) a requirement for intubation; 2) readiness for weaning from mechanical ventilation as defined by recovery from the cause of respiratory failure, a stable hemodynamic status, no requirement for a vasopressor, and no administration of sedative agents or neuromuscular blocking agents > 24 h before enrollment; and 3) readiness for respiration with a T-piece system.

The exclusion criteria were the presence of pneumothorax or ascites, a history of either neuromuscular disease or thoracic surgery, the presence of a tracheostomy tube, and poor image quality. The following baseline characteristics were recorded: age, sex, duration of mechanical ventilation, cause of respiratory failure, body mass index, and laboratory findings before extubation.

### Measurements

In our units, there is no specific (written) criteria by which to determine the best time to start weaning and no formal guidelines for reducing support. In the present study, the patients’ readiness to wean was assessed daily by clinical judgment of the attending intensivist and/or pulmonary physician. The primary physicians were blinded to the ultrasound results. The research team did not play a role in deciding whether a patient was to be extubated.

Exhaled TV, RSBI, VC, PI_MAX_, and diaphragmatic parameters were measured in all participants at the end of a 2-h spontaneous breathing trial with a T-piece and zero pressure support (before extubation). We employed a hand-held Wright respirometer (Ferraris Medical Ltd., Hertford, Hertfordshire, England) to measure minute ventilation (MV). TV was calculated as MV divided by respiratory rate. The RSBI was the ratio between the respiratory rate (breaths/min) and TV (liters) [19]. Measurement of VC (slow VC) was performed in the upright position, after measurement of MV. During measurement of VC, each patient breathed until the end of inspiration, then exhaled to the approximate residual volume. This process was supervised by a pulmonary fellow. PI_MAX_ was measured with a negative inspiratory force meter, which is a calibrated device containing a unidirectional valve, attached to the end of an endotracheal tube (Instrumentation Industries, Inc., Bethel Park, PA, USA). PI_MAX_ was determined from the most negative pressure documented during 20 s of airway occlusion [19].

Transthoracic ultrasonography was performed at the bedside with a SonoSite M-Turbo (SonoSite Inc., Bothell, WA, USA) by a well-trained pulmonary physician. The examination was performed in both B- and anatomical M-modes. All examinations were carried out with patients in the supine position. We obtained diaphragmatic ultrasound values from three consecutive tidal breaths, and the average values were used for analysis.

**Fig. 1:**
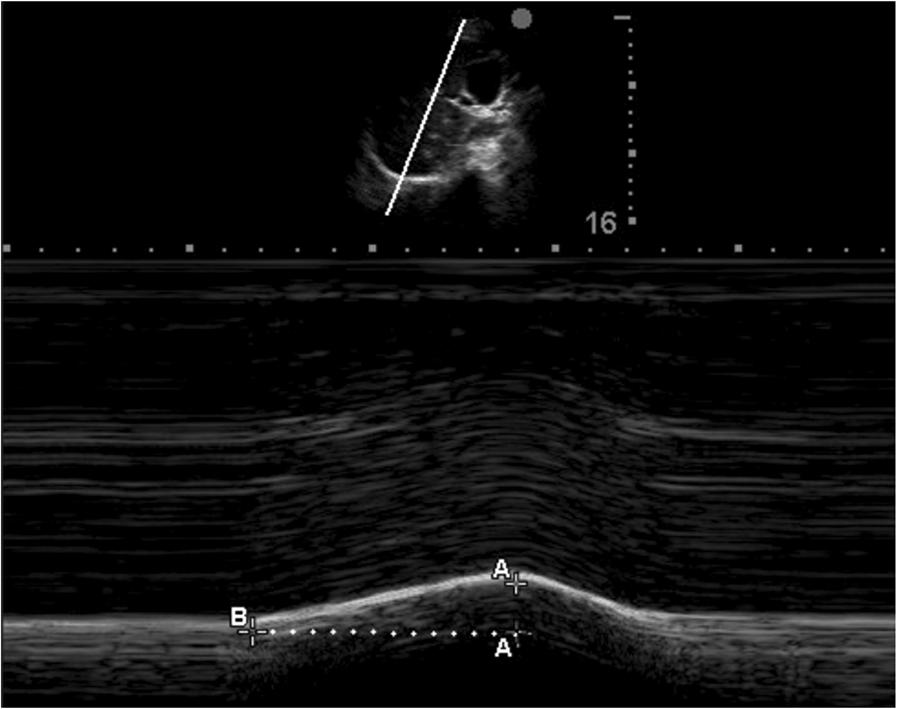
Image showing measurement of the diaphragmatic inspiratory excursion and TPIA_dia_ in M-mode ultrasound. The A-A is the height of diaphragmatic inspiratory excursion. The B-A is the TPIA_dia_. TPIA_dia_, time to peak inspiratory amplitude

The diaphragmatic inspiratory excursion and time to peak inspiratory amplitude of the diaphragm (TPIA_dia_) of each hemidiaphragm (right TPIA_dia_ and left TPIA_dia_) were measured in M-mode using a 1- to 5-MHz ultrasound transducer during tidal breathing (Fig. 1) [20]. The measurement was performed via either a subcostal or intercostal approach in the mid-clavicular line, or in the right or left anterior axillary line. The liver or spleen was identified as a window for each hemidiaphragm. The ultrasound probe was placed in the direction in which the ultrasound beam reached the posterior third of the corresponding hemidiaphragm perpendicularly [15]. During inspiration, the normal diaphragm moved caudally toward the ultrasound transducer, which was recorded as an upward motion of the M-mode tracing. The amplitude of diaphragmatic inspiratory excursion was measured as the point of maximal height of inspiration in the M-mode tracing. The TPIA_dia_ was defined as the time from the beginning of diaphragmatic contraction to the maximal amplitude of diaphragmatic inspiratory excursion as measured from the M-mode tracing.

**Fig. 2:**
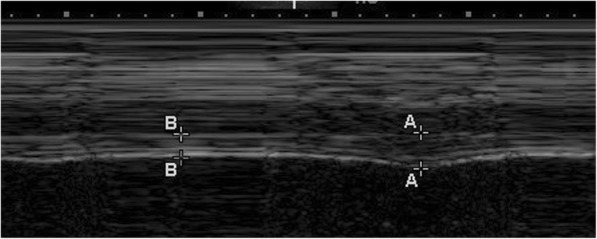
Image illustrating the part of the diaphragm at the area of apposition for measurement of the diaphragmatic thickness (DT). The A-A is the DT at end inspiration, and the B-B is the DT at end expiration

DT was subsequently measured at the zone of apposition [15], which is the area of the diaphragm attached to the rib cage, at both end inspiration and end expiration using a 6–13 ultrasound transducer in M-mode (Fig. 2). The DT difference (DTD) was calculated by subtracting DT at end expiration from DT at end inspiration. The diaphragm thickening fraction (TFdi) was calculated as follows: (DTD)/(DT at end expiration) × 100 [21]. These data were not communicated to the patient care team during the study; they were stored on the hard disk of the ultrasound machine and later exported to files for further verification and analysis. All images were reviewed in terms of image quality by a pulmonary physician and a radiologist (P.T. and T.S.).

### Outcomes

The primary outcome was the diagnostic accuracy of diaphragmatic ultrasound parameters for predicting weaning success. Successful weaning from mechanical ventilation was defined as the ability to tolerate spontaneous breathing for at least 48 h without any level of assisted ventilation. Weaning failure was defined as the requirement for resumption of either invasive or noninvasive mechanical ventilation or reintubation within 48 h after extubation [16].

The secondary outcomes were the association between weaning parameters and diaphragmatic dysfunction [16], reintubation during admission, and reintubation within 48 h after extubation. Ultrasonographic diaphragmatic dysfunction was considered when each hemidiaphragm exhibited either vertical excursion of < 10 mm or the presence of paradoxical movement [16].

### Statistical analysis

The patients were classified into two groups based on the primary outcome. All parameters were compared between the two groups. The unpaired Student’s t-test was used to compare continuous variables, and the chi-square test was used to compare categorical variables. Data are presented as mean ± standard deviation for continuous variables with a normal distribution and as median with interquartile range for variables without a normal distribution.

The variables associated with the primary and secondary outcomes were then analyzed with receiver operating characteristic (ROC) curves to determine their performance. Sensitivity and specificity were also analyzed to determine appropriate cut-off values of TPIA_dia_ and RSBI.

To determine the correlation between diaphragmatic ultrasound values and weaning parameters, we used Pearson’s correlation and presented the results as correlation coefficients. A *P* value of < 0.05 was considered statistically significant. Correlation coefficients of 0.00 to 0.19, 0.20 to 0.39, 0.40 to 0.59, 0.60 to 0.79, and 0.80 to 1.00 were considered to indicate very weak, weak, moderate, strong, and very strong correlations, respectively. We also performed subgroup analysis between patients with and without diaphragmatic dysfunction.

We analyzed inter-operator variability and intra-operator reproducibility of all diaphragmatic ultrasound parameters in 10 cases by two independent operators who were blinded. The former analysis was performed by Pearson’s correlation, and the other was performed with intraclass correlations presented with the correlation coefficient, *P* value, and 95% confidence interval. All statistical analyses were performed using IBM SPSS Statistics for Windows, version 22.0 (IBM Corp., Armonk, NY, USA).

## Results

**Fig. 3:**
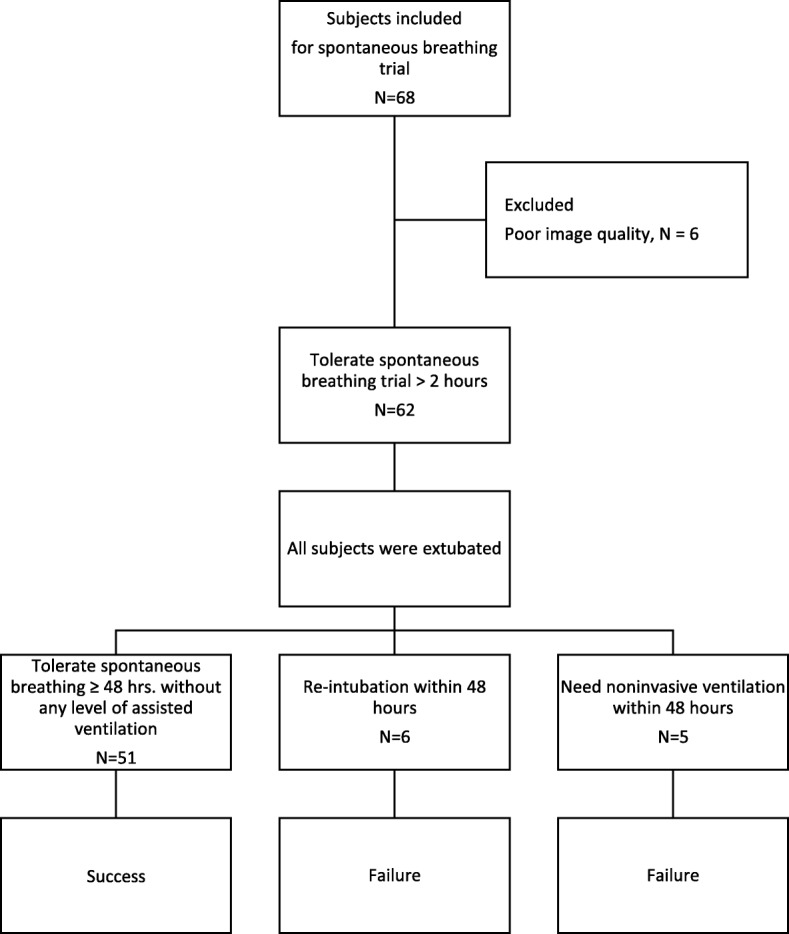
Flow chart showing the number of patients with weaning success, weaning failure, and reintubation

In total, 68 patients were enrolled in our study. In six (9%) patients, however, the examination was not possible or was limited, resulting in poor image quality. These patients were finally excluded. The images of 62 patients were approved for analysis on the basis of quality. The numbers of patients with weaning success and reintubation are shown in Fig. 3. The patients’ baseline characteristics are shown in Table 1. All diaphragmatic parameters obtained from the right and left hemidiaphragm were compared (Table 2), and no difference between the right and left diaphragmatic parameters was seen. Consequently, we presented the ultrasound parameters according to the right hemidiaphragm.

**Table 1:** baseline characteristics

Demographic data	Value
Age (mean ± SD, years)	66.48 ± 16.7
Genders Male (n/%)	38/61
Body mass index (mean ± SD, kg/m^2^)	22.5 ± 6.3
Medical patients (n/%)	42/68
Surgical patients (n/%)	20/32
APACHE II score (mean ± SD)	18.5 ± 7.2
Comorbidity (n/%)	
Hypertension	35/57
Diabetes	21/34
Chronic kidney disease	18/29
Ischemic heart disease	14/23
COPD	6/10
Hypothyroid	5/8
Adrenal insufficiency	3/5
Asthma	2/3
Reason for intubation (n/%)	
Neurological disease	10/16
Septic shock	10/16
Pneumonia	8/13
Heart disease	8/13
COPD exacerbation	4/7
Post-operative conditions	21/34
Laboratory findings (mean ± SD)	
Sodium (mmol/L)	135.9 ± 5.2
Potassium (mmol/L)	3.9 ± 0.59
Bicarbonate (mmol/L)	23.6 ± 4.5
Calcium (mmol/L)	7.9 ± 0.65
Magnesium (mmol/L)	1.97 ± 0.37
Phosphate (mmol/L)	4.6 ± 5.4
Albumin (g/dl)	24.8 ± 5.6
BUN (mg/dl)	29.3 ± 23.9
Creatinine (mg/dl)	1.9 ± 2.3
Hematocrit (%)	31.8 ± 5.5
Vital capacity (mean ± SD, L)	0.97 ± 0.4
Tidal volume (mean ± SD, L)	0.41 ± 0.2
PI_MAX_ (mean ± SD, mmHg)	40.7 ± 16.4
RSBI (mean ± SD, breath•min^− 1^/L)	60.2 ± 33.1
Duration of mechanical ventilation(days) ^a^	2.8(1.1–5.7)
Length of stay (days)	
Intensive care stay^a^	4.8 (2.9–9.6)
Hospital stay^a^	13.3 (7.8–25.9)

*APACHE II* Acute Physiology and Chronic Health Evaluation II, *COPD* chronic obstructive airway disease, *BUN* blood urea nitrogen, *PI*_*MAX*_ Maximum peak inspiratory pressure, *RSBI* Rapid shallow breathing index

^a^present as median (inter quartile range)

**Table 2:** comparison between right and left diaphragmatic parameters

Ultrasonographic parameters	Right	Left	*P* value
Diaphragmatic inspiratory excursion (mm.)	13.5 ± 6.5	13.4 ± 6.2	0.87
TPIA_dia_ (second)	1.2 ± 0.40	1.1 ± 0.39	0.08
Inspiratory DT (mm.)	3.8 ± 1.0	3.8 ± 1.0	0.33
Expiratory DT (mm.)	2.8 ± 0.7	2.8 ± 0. 6	0.52
DTD (mm.)	9.8 ± 4.9	10.1 ± 5.1	0.51
TFdi (%)	36 ± 17	35 ± 14	0.89

*TPIA*_*dia*_ Time to peak inspiratory amplitude of diaphragm, *DT* diaphragmatic thickness, *DTD* Diaphragmatic thickness difference, *TFdi* diaphragm thickening fraction

All data are present as mean ± SD

With the exception of the potassium level, the demographic factors and laboratory findings did not differ significantly between the weaning success and failure groups (Table 3). The mean TPIA_dia_ was significantly higher in the weaning success than failure group (*P* <  0.05). The RSBI during the spontaneous breathing trial was significantly lower in the weaning success than failure group (*P* <  0.01) (Table 3).

**Table 3:** comparison between success group and failure group

Parameters	Weaning success (*n* = 51)	Weaning Failure (*n* = 11)	*P* value	95% CI
Age (mean ± SD, years)	65.90 ± 17.05	69.18 ± 15.45	NS	−7.89 to 14.45
Gender (n/%)				
Female	33/65	5/45		
Male	18/35	6/55	NS	
BMI (mean ± SD, kg/m^2^)	22.63 ± 6.18	21.91 ± 7.66	NS	−5.60 to 4.18
APACHE II score	18.29 ± 7.88	19.55 ± 2.34	NS	−3.58 to 6.08
Reason for intubation (n/%)				
Neurological disease	8/15.7	2/18.2	0.838	0.22 to 6.59
Septic shock	8/15.7	2/18.2	0.838	0.22 to 6.59
Pneumonia	6/11.8	2/18.2	0.623	0.29 to 9.62
Heart disease	6/11.8	2/18.2	0.623	0.29 to 9.62
COPD exacerbation	3/5.9	1/9.1	0.694	0.15 to 17.00
Post-operative conditions	19/37.3	2/18.2	0.305	0.07 to 1.92
TV (mean ± SD, ml.)	420.10 ± 207.48	370.91 ± 137.38	NS	− 82.17 to 180.55
VC (mean ± SD, L.)	1.01 ± 0.44	0.76 ± 0.34	NS	−0.05 to 0.54
RSBI	55.11 ± 25.02	83.84 ± 53.11	P < 0.01	−49.67 to −7.79
PI_MAX_ (mean ± SD, mmHg)	42.31 ± 17.17	32.50 ± 8.05	NS	−1.34 to 20.97
Electrolytes (mean ± SD)				
Sodium (mmol/L)	135.86 ± 5.03	136.00 ± 6.21	NS	−3.63 to 3.35
Potassium (mmol/L)	4.00 ± 0.57	3.46 ± 0.47	P < 0.01	0.17 to 0.92
Bicarbonate (mmol/L)	23.54 ± 4.63	23.81 ± 4.11	NS	−3.29 to 2.75
Magnesium (mmol/L)	1.97 ± 0.37	1.99 ± 0.43	NS	−0.32 to 0.28
Phosphate (mmol/L)	4.92 ± 5.82	3.01 ± 0.56	NS	−2.58 to 6.38
Calcium (mmol/L)	7.88 ± 0.63	8.06 ± 0.83	NS	−0.72 to 0.37
Albumin (g/dl)	25.29 ± 5.34	21.79 ± 6.26	NS	−0.72 to 7.72
Hematocrit (%)	32 ± 6	31 ± 4	NS	−4.79 to 2.58
Diaphragmatic inspiratory excursion (mean ± SD, mm.)				
Right	13.7 ± 5.6	12.4 ± 10.0	NS	−0.31 to 0.56
Left	13.5 ± 5.3	12.8 ± 09.6	NS	−0.35 to 0.48
TPIA_dia_ (mean ± SD, second.)				
Right	1.27 ± 0.38	0.97 ± 0.43	P < 0.05	0.04 to 0.56
Left	1.14 ± 0.37	0.85 ± 0.39	P < 0.05	0.04 to 0.54
DTD (mean ± SD, mm.)				
Right	1.01 ± 0.46	0.84 ± 0.66	NS	−1.61 to 5.00
Left	1.05 ± 0.50	0.80 ± 0.48	NS	−0.84 to 5.82
TFdi (mean ± SD,%)				
Right	36 ± 15	33 ± 24	NS	−15.05 to 7.37
Left	37 ± 14	28 ± 13	NS	−18.16 to 0.65

*COPD* chronic obstructive airway disease, *APACHE II* acute physiology and chronic health evaluation II, *TV* Tidal volume, *VC* vital capacity, *RSBI* rapid shallow breathing index, *PI*_*MAX*_ Maximum peak inspiratory pressure, *TPIA*_*dia*_ time to peak inspiratory amplitude, *DTD* diaphragmatic thickness difference, *TFdi* diaphragm thickening fraction

**Fig. 4:**
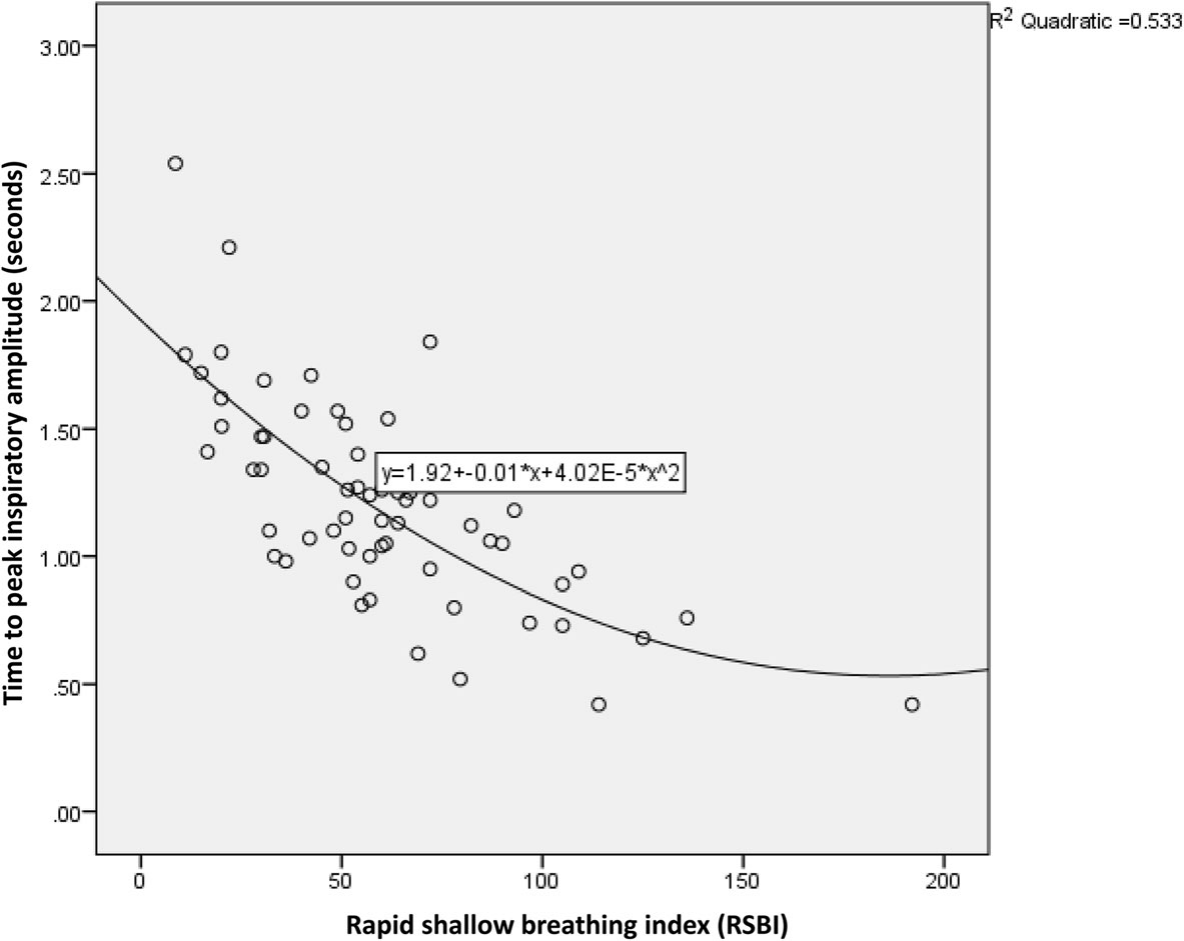
Relationship between the RSBI and TPIA_dia_. RSBI, rapid shallow breathing index; TPIA_dia_, time to peak inspiratory amplitude

Table 4 shows the correlation coefficients between diaphragmatic and conventional parameters. The TPIA_dia_ was significantly correlated with TV and inversely correlated with the RSBI (Fig. 4). There were moderate correlations between TPIA_dia_ and VC, diaphragmatic inspiratory excursion and RSBI, and diaphragmatic inspiratory excursion and TV. The RSBI was inversely related to all diaphragmatic ultrasound parameters. For example, the lower the RSBI, the longer the TPIA_dia_. There was no correlation between PI_MAX_ and any diaphragmatic ultrasound parameters.

**Table 4:** Correlation coefficient among parameters

	TV	VC	RSBI	PI_MAX_
Diaphragmatic inspiratory excursion	0.445*	0.217	−0.435*	0.077
TPIA_dia_	0.639*	0.522*	− 0.703*	0.250
DTD	0.045	−0.017	−0.260^π^	0.166
TFdi	0.001	0.117	−0.218	0.237

*TV* Tidal volume, *VC* vital capacity, *RSBI* rapid shallow breathing index, *PI*_*MAX*_ Maximum peak inspiratory pressure, *TPIA*_*dia*_ time to peak inspiratory amplitude, *DTD* diaphragmatic thickness difference, *TFdi* diaphragm thickening fraction

*P value < 0.01

^π^*P* value < 0.05

Table 5 summarizes the sensitivity, specificity, PPV, and NPV of a TPIA_dia_ of > 0.8 s and an RSBI of < 100 in predicting weaning success.

**Table 5:** Sensitivity, specificity, and negative and positive predictive values of ultrasonography and rapid shallow breathing index for predicting of weaning success

Parameters	Sensitivity	Specificity	Positive predictive value	Negative predictive value	Area under the curve
TPIA > 0.8 s	92.2	45.5	88.7	55.6	0.676
RSBI < 100	96.1	45.5	89.1	71.4	0.659

*TPIA*_*dia*_ time to peak inspiratory amplitude, *RSBI* rapid shallow breathing index

Regarding secondary outcomes, diaphragmatic inspiratory excursion, DTD, and TFdi were significantly lower in patients who underwent reintubation within 48 h. The *P* values were 0.047, 0.021, and 0.028, and the areas under the ROC curves were 0.716, 0.805, and 0.784, respectively. A diaphragmatic inspiratory excursion cut-off value of 12.85 mm provided a sensitivity of 83% and specificity of 55% for predicting reintubation within 48 h. The PPV and NPV of a diaphragmatic inspiratory excursion value of < 12.85 mm were 17 and 97%, respectively. A DTD of < 0.65 mm predicted reintubation within 48 h with a sensitivity and specificity of 83 and 77%, respectively. The PPV of a DTD of < 0.65 mm was 28%, and the NPV was 98%.

Nineteen of 62 (31%) patients had diaphragmatic dysfunction according to the criteria established by Kim et al. [16]. Patients who developed diaphragmatic dysfunction had a significantly higher RSBI and lower TPIA_dia_, DTD, TFdi, TV, and PI_MAX_ (Table 6). The areas under the ROC curves of RSBI, TPIA_dia_, DTD, TFdi, TV, VC, and PI_MAX_ for predicting diaphragmatic dysfunction were 0.720, 0.722, 0.697, 0.762, 0.700, 0.570, and 0.653, respectively.

**Table 6:** a comparison between groups of diaphragmatic dysfunction and non- diaphragmatic dysfunction

Parameters	Diaphragmatic dysfunction (*n* = 19)	Non-diaphragmatic dysfunction (*n* = 43)	*P* value	95% CI
TV (mean ± SD, ml.)	318.68 ± 83.46	452.33 ± 218.34	< 0.05	29.87 to 237.41
VC (mean ± SD, L.)	838.89 ± 263.77	1019.30 ± 480.77	NS	−60.93 to 421.76
RSBI	79.53 ± 40.24	51.67 ± 25.63	< 0.01	−44.81to −10.92
PI_MAX_ (mean ± SD, mmHg)	33.72 ± 11.08	43.63 ± 17.45	< 0.05	0.98 to 18.83
TPIA_dia_ (mean ± SD, second)	0.99 ± 0.33	1.31 ± 0.39	< 0.01	0.12 to 0.53
DTD (mean ± SD, mm.)	0.75 ± 0.50	1.08 ± 0.47	< 0.05	0.66 to 5.92
TFdi (mean ± SD,%)	25 ± 12	40 ± 16	< 0.01	6.79 to 23.69

*TV* Tidal volume, *VC* vital capacity, *RSBI* rapid shallow breathing index, *PI*_*MAX*_ Maximum peak inspiratory pressure, *TPIA*_*dia*_ time to peak inspiratory amplitude, *DTD* diaphragmatic thickness difference, *TFdi* diaphragm thickening fraction

The parameters that predicted reintubation during admission were PI_MAX_, RSBI, VC, and TPIA_dia_. The areas under the ROC curves were 0.725, 0.652, 0.735, and 0.710, respectively.

The intra-operator reproducibility analysis of TPIA in the two operators showed an intraclass correlation coefficient of 0.97 (95% confidence interval, 0.90–0.99; *P* <  0.001) and 0.92 (95% confidence interval, 0.70–0.98; P <  0.001), respectively. The inter-operator variability analysis between the two operators revealed a Pearson correlation coefficient ® of 0.95 (P <  0.001). The remaining parameters were analyzed as shown in Additional file 1: Table S1.

## Discussion

Ultrasonography provides some advantages as a noninvasive diagnostic tool in critically ill patients, including availability at the bedside and avoidance of radiation hazards. The role of ultrasound in the assessment of diaphragm function has been studied [16] with the rationale that the diaphragm plays a crucial role in respiratory muscle endurance. However, data concerning its usefulness as a weaning predictor are limited [16, 17, 21–23]. We investigated the diagnostic performance of diaphragmatic function parameters assessed by ultrasonography to predict the success of weaning from mechanical ventilation.

The main findings of this study can be summarized as follows. 1) The prevalence of ultrasonographic diaphragmatic dysfunction was 31%. 2) The TPIA_dia_ was a parameter that could be used to predict successful weaning from mechanical ventilation and had a strong correlation with the RSBI. 3) With respect to secondary outcomes, the diaphragmatic inspiratory excursion, DTD, and TFdi could predict reintubation within 48 h. 4) There was no difference between the right and left diaphragmatic parameters.

Diaphragmatic dysfunction is a common condition in the ICU and is associated with prolonged weaning from mechanical ventilation and weaning failure [16]. Jiang et al. [23] hypothesized that displacement of the liver/spleen as measured by ultrasonography can represent movement of the hemidiaphragms. They demonstrated that using a cutoff value of 1.1 cm can predict successful extubation [23]. Our study showed that the prevalence of diaphragmatic dysfunction in the medical and surgical ICU was 31%, which is in line with that reported by Kim et al. [16], who demonstrated that 29% of patients hospitalized in the medical ICU developed diaphragmatic dysfunction.

Some previous investigators have performed only right hemidiaphragm ultrasound in the ICU because the acoustic window provided by the liver makes it easier to measure the parameters on this side [17, 21]. In the present study, we completed both right and left hemidiaphragm measurements without any difficulty. Nevertheless, we found that there was no significant difference between the right and left diaphragmatic parameters (Table 2). Therefore, measurement of the right hemidiaphragm may be more practical in clinical practice.

To the best of our knowledge, this is the first study to compare the performance of various diaphragmatic ultrasound-derived parameters with respect to weaning outcomes in critically ill patients. We also proposed a parameter, the TPIA_dia_, to predict successful weaning from mechanical ventilation. We found that patients with a longer TPIA_dia_ tended to have more successful weaning from mechanical ventilation, and we found a strong correlation between the TPIA_dia_ and RSBI. This correlation suggests the presence of a relationship between TPIA_dia_ and diaphragmatic strength. Physiologically, the strength and endurance of the diaphragm are not similar. Diaphragmatic strength refers to the capacity of the diaphragm to generate force, which is dependent on many factors such as diaphragm contractile activation, excitation–contraction coupling, central drive, nerve conductance, and neuromuscular transmission [24]. Endurance is defined as the ability of the diaphragm to sustain force over time. Our investigation suggests that the TPIA_dia_ is associated with the strength of the diaphragm rather than its endurance.

In general, a more extended TPIA_dia_ increases the diaphragmatic amplitude. Therefore, both parameters should display similar results. Although the diaphragmatic inspiratory excursion could predict reintubation, it could not predict weaning success. This result suggests that the diaphragmatic inspiratory excursion represents the endurance of the diaphragm rather than its strength. Why the inspiratory excursion showed a different result from the TPIA_dia_ is not clear. However, we hypothesize that an individual patient may have his or her own inspiratory time and diaphragmatic amplitude for a required TV. This idea is supported by the relationship between the product of pressure created by respiratory muscle (Pmus) and the electrical activity of the diaphragm (EAdi) measured in neutrally adjusted ventilatory assist mode, as demonstrated in a study by Bellani et al. [25]. The authors showed a different Pmus/EAdi ratio in each patient and indicated that the patients had their own ratios. Consequently, we anticipate different TPIA_dia_/diaphragmatic inspiratory excursion ratios among our patients, and they may have specific ratios of their own. Thus, we found a correlation coefficient of only 0.5 between the TPIA_dia_ and diaphragmatic inspiratory excursion in an additional analysis. However, we need to further prove this hypothesis.

The TPIA_dia_ showed an association with the success of breathing spontaneously without any assisted ventilation, whereas the DTD and TFdi were related to reintubation among the patients in our study. This can be explained by the fact that the DTD represents the endurance of the diaphragm, which is a major factor leading to reintubation. However, we could not explain why the diaphragmatic inspiratory excursion provides a similar result with respect to thickness difference parameters. The transdiaphragmatic pressure–time product needs further determination.

We found no correlation between PI_MAX_ and TPIA_dia_ or other diaphragmatic ultrasound parameters. PI_MAX_ is a well-known index of respiratory muscle strength. However, some investigators have found that PI_MAX_ might be unreliable among patients in the medical ICU [26]. The most objective diagnostic test of respiratory muscle weakness is determination of the transdiaphragmatic pressure in response to phrenic nerve stimulation (PdiTw). Supinski et al. [27] investigated the correlation between PdiTw and PI_MAX_. They found that when plotting PI_MAX_ against PdiTw, significant scatter was present between the two values, suggesting the unreliability of predicting PdiTw from the determination of PI_MAX_. Nevertheless, each value is a predictor of both mortality and the duration of mechanical ventilation. This is in line with our study with respect to the fact that no correlation was found between PI_MAX_ and any diaphragmatic ultrasound parameters. The principles of measurement between these parameters are different. The PdiTw is affected by stimulation of the phrenic nerves, and diaphragmatic ultrasound evaluates mainly the diaphragm, whereas PI_MAX_ evaluates the inspiratory pressure by the contraction of all inspiratory muscles. Moreover, the diaphragmatic ultrasound examination and measurement of PdiTw were performed during submaximal breathing while measurement of PI_MAX_ was performed to assess the highest voluntary inspiratory muscle pressure generation.

Although the areas under the ROC curve of TPIA_dia_ and the other ultrasound criteria were rather low in our study, they were at least equal to or better than the RSBI, the well-known criteria used in clinical practice. The area under the curve of the ultrasound criteria used to predict the weaning outcome varies from 0.68 to 0.79 among different studies [16, 22]. As evidenced by the quite low area under the curve of these ultrasound criteria indices, weaning failure depends on several clinical factors; for example, it can result not only from diaphragmatic dysfunction or critical illness myopathy but also excess mechanical load, impairment of respiratory mechanics, cardiovascular dysfunction, or an inability to clear secretions. Thus, a single diaphragmatic index might not be a perfect predictor, as shown in a study by Tenza-Lozano et al. [28]. They demonstrated that the area under the ROC curve of TFdi as a single predictor was 0.71. The performance of TFdi combined with lung ultrasound improved the prediction of weaning success with an AUC of 0.83, which supports our above-mentioned hypothesis [28]. Furthermore, we enrolled many patients of advanced age in the present study. The causes of weaning failure in older patients are multifactorial [29], and few specific parameters are available to predict weaning outcomes in this population [30]. Therefore, diaphragmatic parameters should be combined with other parameters for predicting weaning outcomes.

Since 1991, the RSBI has been used to predict weaning outcomes [19]. It exhibits the best performance compared with the CROP index, PI_MAX_, and MV. However, the isolated RSBI may not be precise enough to predict weaning outcomes in patients undergoing prolonged mechanical ventilation [31]. The RSBI is a weaning predictor that measures the change in volume generated by all respiratory muscles while not specifically measuring the diaphragmatic contribution. A study by Kuo et al. [32] showed better performance of the RSBI at the 2-h time point of a spontaneous breathing trial than at the beginning. Consequently, the initial RSBI may not allow for proper assessment of patients with impaired diaphragmatic endurance but may be appropriate for assessment of impaired diaphragmatic strength. We did not find that the RSBI was associated with reintubation within 48 h after extubation in the present study.

Our study has some limitations. We enrolled both medical and postoperative patients in this study. Therefore, we cannot apply the results to specific diseases, such as chronic obstructive pulmonary disease. Further studies should be conducted to evaluate the role of TPIA_dia_ in specific diseases. TPIA_dia_ provided good sensitivity but low specificity for predicting successful weaning from mechanical ventilation, which means that there were some false-positives at low TPIA_dia_ values. In other words, a given patient with a TPIA_dia_ of ≤0.8 s could still have weaning success with an NPV of 56. However, a positive result that predicts weaning failure does not mean that the patient would have delayed weaning, but should be carefully evaluated during the weaning and extubation periods. Therefore, TPIA_dia_ should be interpreted with caution and should be combined with other parameters. A validation cohort study should be conducted to confirm the results of our study. Furthermore, we analyzed the predictors of reintubation during hospital admission, and several uncontrolled variables could have interfered with the outcome (e.g., amount of secretion, consciousness level, or deterioration of the hemodynamic status).

## Conclusions

Among diaphragmatic parameters, the TPIA_dia_ exhibited good performance in predicting weaning outcomes and had a strong correlation with the RSBI. This study demonstrated a trend toward focusing on the TPIA_dia_, rather than diaphragmatic inspiratory excursion, as a predictor of weaning from mechanical ventilation.

## Additional file

Additional file 1: **Table S1.** Inter-operator variability and intra-operator reproducibility of diaphragmatic ultrasound parameters. *Analyzed by intraclass correlation and presented as intraclass correlation coefficient. **Analyzed by Pearson’s correlation and presented as correlation coefficient ®. All parameters exhibited a significant correlation (*P* <  0.001). TPIA_dia_; Time to peak inspiratory amplitude of diaphragm, DT; diaphragmatic thickness. (DOCX 13 kb)

## Abbreviations

DT: Diaphragmatic thickness
DTD: Diaphragmatic thickness difference
ICU: Intensive care unit
MV: Minute ventilation
NPV: Negative predictive value
PdiTw: Transdiaphragmatic pressure in response to phrenic nerve stimulation
PI_MAX_: Maximum peak inspiratory pressure
PPV: Positive predictive value
ROC: Receiver operating characteristic
RSBI: Rapid shallow breathing index
TFdi: Diaphragm thickening fraction
TPIA_dia_: Time to peak inspiratory amplitude of diaphragm
TV: Tidal volume
VC: Vital capacity
