# Supplementary material for: Diaphragmatic parameters by ultrasonography for predicting weaning outcomes
Source: BMC Pulm Med. 2018 Nov 23;18:175. doi: 10.1186/s12890-018-0739-9 (PMC6251135; doi:10.1186/s12890-018-0739-9)
Supplement: Supplementary file 1 — Table S1. Inter-operator variability and intra-operator reproducibility of diaphragmatic ultrasound parameters. *Analyzed by intraclass correlation and presented as intraclass correlation coefficient. **Analyzed by Pearson’s correlation and presented as correlation coefficient ®. All parameters exhibited a significant correlation (P < 0.001). TPIAdia; Time to peak inspiratory amplitude of diaphragm, DT; diaphragmatic thickness. (DOCX 13 kb) [file 12890_2018_739_MOESM1_ESM.docx]

**Table S1** Inter- operator variability and intra- operator reproducibility of diaphragmatic ultrasound parameters

| Variable | Intra-operator* | | Inter-operator** |
| --- | --- | --- | --- |
|  | Operator A | Operator B |  |
| Diaphragmatic inspiratory excursion (mm.) | 0.99 | 0.99 | 0.99 |
| TPIA_dia_ (seconds) | 0.97 | 0.92 | 0.95 |
| Inspiratory DT (mm.) | 0.99 | 0.99 | 0.93 |
| Expiratory DT (mm.) | 0.99 | 0.99 | 0.94 |

*Analyzed by intraclass correlation and presented as intraclass correlation coefficient

**Analyzed by Pearson’s correlation and presented as correlation coefficient (r)

All parameters exhibited a significant correlation (P<0.001).

TPIA_dia_ ; Time to peak inspiratory amplitude of diaphragm, DT; diaphragmatic thickness
